# Unusual presentations of Churg Strauss syndrome in paediatrics: a tale of 2 masses from one centre experience

**DOI:** 10.1186/1546-0096-9-S1-P96

**Published:** 2011-09-14

**Authors:** E Baildam, C Pain, F McErlane, G Cleary, M Beresford, L McCann

## Background

To highlight unusual presentations of Churg Strauss Syndrome (CSS) in children.

## Aim

Raising clinical awareness of atypical presentations.

## Methods

Case note review and literature search.

## Results

### Case 1

A 15 year old girl with worsening asthma and rhinitis presented with a persistent 10x6 cm breast lump 6 months after starting Montelukast. Chest X-rays and CT scan were normal, maxillary sinuses congested, with absolute eosinophilia of 2.4 x 10^9^/l, ESR normal, no proteinuria). On needle biopsy, dilated ducts contained necrotic eosinophilic debris, intense infiltrate of eosinophils and plasma cells without granulomatous infiltration or vasculitis. Despite withdrawing Montelukast the mass persisted but treatment with steroids was successful.

### Case 2

A 14 year old girl presented with worsening asthma associated with malaise, weight loss, an intermittent vasculitic rash and abdominal pain. She had a large right ventricular mass with pericardial effusion and endocardial impairment, pulmonary infiltrates, microscopic haematuria, vasculitic changes on cerebral MRI, a raised eosinophil count (6.16x10^9^), anaemia, ESR 114 and positive pANCA (non PR3 / MPO). A good clinical response to treatment with steroids, cyclophosphamide (IV) and immunoglobulin is currently maintained on MMF and low dose steroid with anticoagulation (warfarin). Her cardiac mass has not regressed, but remained static, most likely representing chronic endocardial fibrosis.

## Conclusion

These cases illustrate atypical presentations of CSS in children. Eosinophilic mastitis may be idiopathic, parasitic or part of CSS - reported following treatment with Montelukast but never with eosinophilic mastitis. Cardiac masses in CSS have been reported occasionally in adults, but not previously seen in children.

